# Precision nutrition in Asian populations: a Multi-omics review of mechanisms, biomarkers, and implementation pathways

**DOI:** 10.1017/jns.2026.10110

**Published:** 2026-07-09

**Authors:** Minjoo Kim

**Affiliations:** Department of Food and Nutrition, Hannam University, Daejeon, Republic of Korea

**Keywords:** Diet–metabolism interactions, Genomics, Metabolic biomarkers, Metabolic diseases, Metabolomics, Precision nutrition

## Abstract

The rapid expansion of omics technologies has created new opportunities to understand inter-individual variations in metabolic responses to diet. Such advances are particularly relevant for Asian populations, which exhibit distinct metabolic characteristics, including increased visceral adiposity, reduced β-cell reserves, and heightened susceptibility to type 2 diabetes at lower BMI levels, compared to Western populations. This review synthesizes the current evidence on metabolomic and genomic biomarkers associated with metabolic health in Asians and outlines the mechanistic pathways through which diet influences these biomarkers. Metabolomic signatures, such as lysophosphatidylcholines, micronutrient-derived metabolites, amino acid profiles, and oxidative stress indicators, have demonstrated strong potential for the early detection of metabolic dysfunction. In addition, carbohydrate-related markers of glycemic excursions, microbiome-derived metabolites, and diet-responsive fatty acid profiles may help capture the heterogeneity in postprandial regulation and diet responsiveness. Genetic variants enriched in Asian populations, including *TMEM182*- and *NPC1L1*-related polymorphisms, further modulate lipid metabolism, adipogenesis, and glycemic regulation. We also highlighted *β*-cell and nutrient-handling loci (e.g. *KCNQ1*, *TCF7L2*, S*LC30A8*, *FUT2/6*, *BCMO1*, and *FADS1/2*) as mechanistic anchors for biologically stratified dietary personalization. We discuss nutrient–metabolite interactions – particularly those involving dietary fibre and legumes – within culturally patterned Asian diets and highlight culturally consistent dietary strategies supported by multi-omics evidence. Finally, we propose a translational framework for implementing precision nutrition in Asia, emphasizing analytical standardization, clinician training, digital health integration, and equity considerations. Together, these insights underscore the potential of multi-omics approaches to inform individualized dietary recommendations and improve metabolic health across diverse Asian populations.

## Graphical abstract

**Figure:**
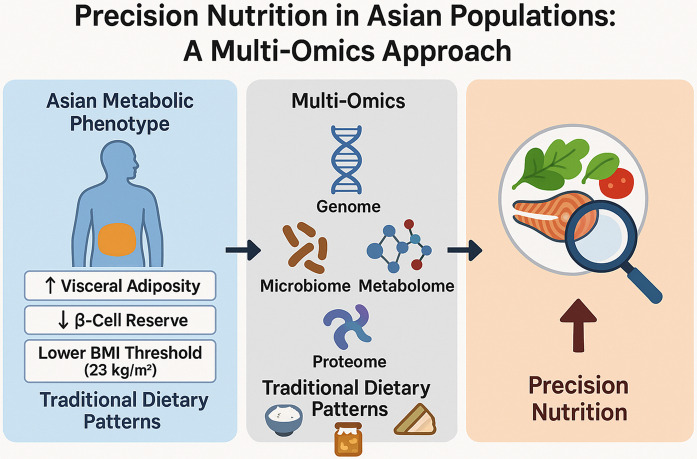

## Introduction

Traditional population-level dietary guidelines, largely derived from Western epidemiological and clinical research, have substantially contributed to global public health.^(1,2)^ However, these guidelines implicitly assume that the physiological responses to dietary components are broadly uniform across populations.^(3,4)^ Growing evidence challenges this assumption, demonstrating that metabolic responses to macronutrient composition, micronutrient quality, and overall dietary patterns differ considerably according to genetic architecture, gut microbiome composition, habitual diet, lifestyle environments, and cultural factors.^(5–7)^ These biological and environmental determinants shape postprandial glucose excursions, lipid handling, inflammatory pathways, and metabolic flexibility, all of which critically influence long-term metabolic health outcomes.^(8,9)^

The advent of omics technologies has transformed our understanding of interindividual variability.^(10,11)^ Rapid advances in genomics, metabolomics, proteomics, and metagenomics have enabled the high-resolution characterization of molecular pathways linking diet to health outcomes.^(5,7,12)^ Omics profiling has revealed nutrient-specific metabolic reactions, diet-sensitive gene–environment interactions, and early biochemical perturbations that precede clinically detectable diseases.^(8,9,13)^ These tools provide unprecedented opportunities to identify individuals who may benefit from targeted dietary strategies and to refine public health recommendations based on biological responsiveness.^(1,3)^

These advances are particularly relevant for Asian populations, who face a disproportionately high burden of metabolic diseases despite having a lower average BMI than Western populations.^(7,14,15)^ East Asians typically exhibit greater visceral adiposity, reduced β-cell reserve, and heightened insulin resistance at comparatively lower BMI thresholds.^(4,7,15)^ South Asians experience an even earlier onset of cardiometabolic risk, frequently driven by high hepatic fat sensitivity, impaired mitochondrial oxidation, and carbohydrate-dominant dietary patterns.^(16,17)^ Southeast Asian populations show additional variability, reflecting diverse dietary traditions, food processing methods, and environmental exposures.^(18)^

Asia’s exceptional dietary diversity – from rice-centred patterns in Northeast and Southeast Asia to lentil-, wheat-, and millet-based diets in South Asia – contributes to population-specific metabolic signatures.^(11,18)^ Likewise, genomic studies have identified Asian-enriched variants affecting lipid metabolism, adipogenesis, cholesterol transport, and glucose regulation.^(17,19–23)^ These genetic and cultural factors underscore the importance of population-specific evidence in guiding precision nutrition strategies.

In this review, we synthesize current metabolomic and genomic evidence from Asian populations, describe nutrient–metabolite mechanisms relevant to dietary guidance, and outline a translational framework for implementing precision nutrition in diverse Asian healthcare and community settings.

## Biological basis for precision nutrition in Asians

Developing precision nutrition strategies tailored to Asian populations requires a detailed understanding of their distinct metabolic and physiological characteristics. Compared with Western populations, Asian populations often exhibit a constellation of traits that increase susceptibility to obesity, metabolic syndrome, and type 2 diabetes.^(4,7)^ These traits include increased visceral adiposity, altered adipokine secretion, lower lean mass, reduced metabolic flexibility, and diminished β-cell functional reserve.^(4,7,15)^

### Visceral adiposity and inflammatory signalling

Even at BMI levels considered normal by Western standards, many Asians accumulate a greater proportion of visceral adipose tissue.^(7,18)^ Visceral adipocytes are metabolically active and are characterized by elevated lipolytic activity and high secretion of pro-inflammatory cytokines, such as tumour necrosis factor-alpha (TNF-α) and interleukin-6 (IL-6).^(24,25)^ These cytokines impair the phosphorylation of insulin receptor substrate-1 (IRS-1) and disrupt downstream phosphatidylinositol 3-kinase (PI3K)–protein kinase B (Akt) signalling, initiating early insulin resistance and promoting hepatic gluconeogenesis.^(25,26)^ Elevated circulating free fatty acids (FFAs) from visceral lipolysis further exacerbate hepatic lipid accumulation and impair muscle glucose uptake.^(26,27)^

### Reduced β-cell reserve and glycemic vulnerability

Multiple studies have indicated that East and South Asians have lower β-cell compensatory capacity, rendering them more vulnerable to postprandial hyperglycaemia when exposed to high-glycemic diets.^(4,16)^ Declines in early-phase insulin secretion appear earlier and more prominently than in Western populations,^(16)^ suggesting a pathophysiological trajectory characterized by impaired secretion rather than insulin resistance alone.^(28)^

### Genetic susceptibility and pathway modulation

Genetic architecture contributes substantially to the distinct metabolic vulnerability observed in many Asian populations. In addition to previously described Asian-enriched variants, such as polymorphisms in *TMEM182, NPC1L1, APOA5, CETP*, and inflammatory pathway genes,^(19–23)^ multiple loci robustly associated with type 2 diabetes and glycemic traits further clarify the biological underpinnings of cardiometabolic risk in this region. Variants in *KCNQ1, TCF7L2*, and *SLC30A8*, repeatedly replicated in East Asian and multi-ancestry genome-wide association studies (GWASs), are particularly informative in this context.^(29–31)^ Their utility in Asian cohorts is further shaped by ancestry-related differences in allele frequencies and linkage disequilibrium, which may affect effect-size transferability from European studies.

These loci underscore the prominent contribution of impaired β-cell function to diabetes susceptibility at lower BMI levels in many Asian groups. *KCNQ1* has demonstrated strong and reproducible associations with type 2 diabetes in East Asian populations and is thought to influence insulin secretion through modulation of β-cell electrophysiology and incretin-related pathways.^(29)^ *TCF7L2*, one of the most consistently replicated genetic signals for type 2 diabetes across ancestries, affects incretin action and insulin secretion, thereby contributing to postprandial glycemic dysregulation.^(30)^ *SLC30A8*, which encodes the β-cell zinc transporter ZnT8, plays a central role in insulin granule formation and glucose-stimulated insulin secretion; functional variation at this locus may modify β-cell resilience under metabolic stress.^(31)^ In populations characterized by limited β-cell compensatory capacity, these variants may amplify glycemic vulnerability when dietary patterns are dominated by refined carbohydrates and high glycemic load.

In addition to glycemic regulation, loci related to micronutrient handling and lipid metabolism provide additional mechanistic anchors for population-tailored precision nutrition. Variants in *FUT2* and *FUT6* influence intestinal glycosylation patterns and host–microbiome interactions, with downstream effects on nutrient absorption, microbial metabolite production, and inflammatory signalling.^(32)^ Given the marked regional diversity in fermented foods, fibre sources, and carbohydrate staples across Asia, such variation may contribute to interindividual differences in postprandial metabolic responses. *BCMO1*, which regulates the conversion efficiency of β-carotene to retinoids, may alter retinoid-mediated transcriptional pathways involved in lipid oxidation and glucose metabolism.^(33)^ In dietary contexts where provitamin A carotenoids constitute a major source of vitamin A, genetic variability in *BCMO1* could meaningfully influence metabolomic profiles and metabolic risk trajectories.

Similarly, polymorphisms in the fatty acid desaturase cluster (FADS1/2) affect endogenous long-chain polyunsaturated fatty acid (PUFA) biosynthesis, circulating lipidomic signatures, and inflammatory tone.^(34)^ Because dietary fat composition varies considerably across Asian regions – ranging from marine-derived n−3 fatty acids to seed- and palm-oil–based fats – *FADS*-related variation may modify cardiometabolic responses to fat quality and n−3/n−6 balance, supporting a genotype-informed approach to dietary fat modulation.

Translationally, these genetic determinants gain clinical relevance when integrated with dynamic omics-derived intermediate phenotypes, including postprandial glycemic excursions assessed by continuous glucose monitoring (CGM), lipidomic profiles reflecting PUFA balance, and biomarkers of oxidative stress.^(5–7,10)^ Genotype-informed risk stratification may therefore help identify biologically coherent responder subgroups. For example, individuals with elevated β-cell–related genetic susceptibility (e.g. *KCNQ1*-, *TCF7L2*-, or *SLC30A8*-implicated profiles) may derive particular benefit from dietary strategies emphasizing glycemic load reduction and improved carbohydrate quality. Conversely, those carrying variants affecting PUFA biosynthesis (*FADS1/2*) or nutrient absorption and metabolism (e.g. *FUT2/6, BCMO1*) may warrant more precise modulation of fatty acid composition or carotenoid-rich food intake. Such integrative approaches strengthen the mechanistic and translational foundation of precision nutrition strategies tailored to heterogeneous Asian populations.

**Figure 1.**
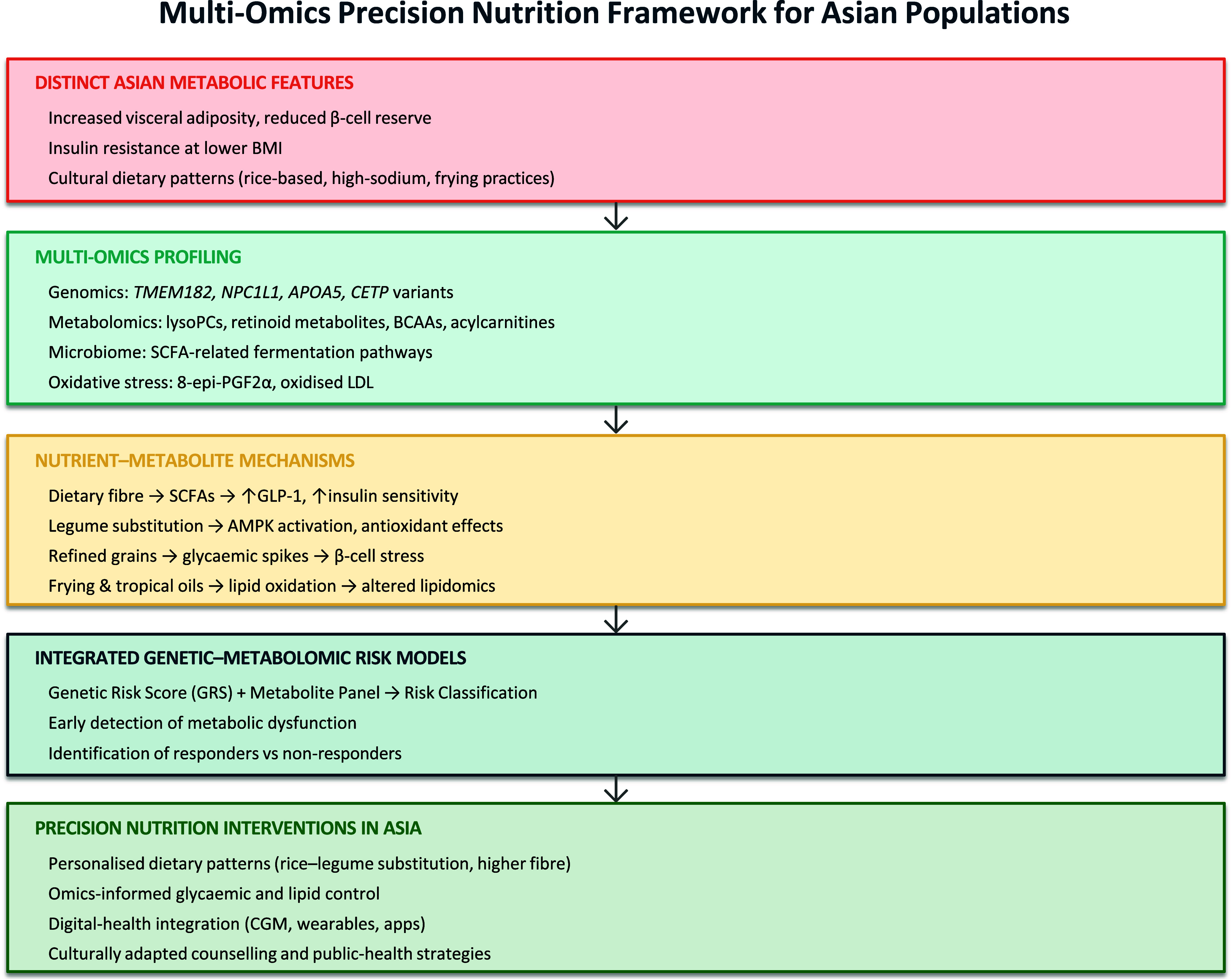
Figure 1 long description. Multi-omics precision nutrition framework for Asian populations. Asian populations exhibit distinct metabolic features, including increased visceral adiposity, reduced β-cell reserve, and culturally specific dietary patterns. Multi-omics profiling (genomics, metabolomics, microbiome, and oxidative stress markers) captures these traits and their responses to diet. The integration of nutrient–metabolite mechanisms and genetic–metabolomic risk models enables the stratification of individuals into differential risk and response groups, supporting culturally adapted precision nutrition interventions in Asian healthcare and community settings.

Collectively, these genetic determinants illustrate how population-specific variation in β-cell function, micronutrient handling, and lipid metabolism can shape metabolic responses to diet in Asian populations. The integration of genotype information with intermediate omics phenotypes provides a mechanistic basis for developing biologically stratified precision nutrition strategies, as summarized in Figure 1.

## Metabolomic biomarkers in Asian populations

Metabolomics provides a dynamic representation of an individual’s biochemical state, reflecting recent dietary intake, metabolic stress, microbiome activity, and environmental exposure.^(9,10,13,35)^ This sensitivity to short- and medium-term metabolic fluctuations makes metabolomic profiling particularly valuable for investigating the risk of metabolic diseases in Asian populations.

### Lipid-derived metabolites and oxidative stress

A consistent finding across Asian metabolomics studies is the elevation of lipid-derived metabolites, particularly lysophosphatidylcholines (lysoPCs).^(36–38)^ LysoPCs are generated through phospholipase A_2_–mediated cleavage of phosphatidylcholines and serve as biomarkers of oxidative stress, membrane turnover, and inflammatory activation.^(9,37,39)^ In multiple Korean cohorts, metabolically unhealthy overweight individuals exhibited elevated lysoPC concentrations, higher levels of F_2_-isoprostanes, and reduced adiponectin levels.^(22,36)^ Mechanistically, lysoPCs impair mitochondrial efficiency, promote endothelial dysfunction, and activate proinflammatory cytokine pathways, contributing to early insulin resistance and increased vascular stress.^(37–40)^

### Carbohydrate-related metabolites and glycemic-excursion markers

Carbohydrate-related metabolites can provide sensitive readouts of postprandial dysglycemia, which is particularly relevant in Asian settings, where refined grains and high glycemic-load staples remain common, and β-cell reserve may be comparatively limited. In addition to fasting glucose and HbA1c, 1,5-anhydroglucitol (1,5-AG) is a well-validated short-term marker of glycemic excursions because it decreases when plasma glucose exceeds the renal threshold, capturing postprandial spikes that may be missed by longer-term averages.^(41)^ Evidence from East Asian clinical studies further supports its usefulness in reflecting postprandial hyperglycaemia and glycemic variability.^(42,43)^ Additional markers linked to early glycemic stress and reduced metabolic flexibility include lactate, α-hydroxybutyrate (α-HB), and plasma mannose, which have been associated with insulin resistance and incident type 2 diabetes in prospective analyses.^(44–46)^ Reactive dicarbonyls (e.g. methylglyoxal and 3-deoxyglucosone) and polyol-pathway metabolites (e.g. sorbitol and fructose) may also indicate carbonyl/redox stress under sustained hyperglycaemia.^(47,48)^ Integrating excursion-sensitive biomarkers (e.g. 1,5-AG) with intermediary metabolism and glycation/redox-stress readouts can help identify ‘carbohydrate responders’ and prioritize interventions targeting carbohydrate quality and meal structure to blunting glucose peaks.

### Microbiome-related metabolites

Microbiome-derived metabolites are particularly informative in Asia because fermented-food practices and heterogeneous fibre sources can reshape gut microbial ecology and downstream metabolite profiles.^(49)^ SCFAs – including acetate, propionate, and butyrate – are produced through microbial fermentation of dietary fibre and can influence host metabolism via host–microbe signalling and enteroendocrine pathways.^(50,51)^ In parallel, trimethylamine N-oxide (TMAO), generated from microbial metabolism of dietary choline- and carnitine-containing substrates followed by hepatic oxidation, has been reported to be associated with adverse cardiovascular outcomes in experimental models and prospective human studies, including growing evidence from Asian cohorts.^(52–54)^ In addition, bile acid pools are increasingly recognized as diet- and microbiome-sensitive endocrine signals through receptors such as the farnesoid X receptor (FXR) and Takeda G-protein–coupled receptor 5 (TGR5), which regulate lipid and carbohydrate metabolism and inflammatory tone, making them attractive candidates for targeted metabolite panels in precision nutrition.^(55,56)^ To avoid redundancy, detailed dietary determinants (e.g. specific fibres and fermented foods) are discussed further in the dietary-mechanisms section below.

### Micronutrient-related biomarkers within metabolomics

Micronutrient-related biomarkers can be integrated into metabolomic panels or assayed alongside them to reflect nutrient status that is clinically actionable and regionally relevant, particularly in Asia, where dietary sources and food environments vary substantially across subregions. Among micronutrients, vitamin A derivatives – particularly retinal and retinoic acid – provide a compelling example of nutrient-linked biochemistry that can inform early metabolic risk stratification; in Korean adults, they have emerged as predictive biomarkers.^(57)^ These metabolites regulate hepatic lipid oxidation, gluconeogenesis, adipocyte differentiation, and insulin sensitivity through RAR/RXR-mediated transcriptional control.^(58,59)^ Elevated retinoid metabolite levels predict the progression from impaired fasting glucose to type 2 diabetes more accurately than conventional markers, such as fasting glucose or HbA1c.^(57)^ When combined with oxidative stress markers such as oxidized LDL and 8-epi-prostaglandin F_2α_ (8-epi-PGF_2α_), retinoid profiles offer a sensitive readout of metabolic stress that precedes clinical deterioration.^(22,36,57)^

In addition to vitamin A, vitamin B_12_-related biomarkers may be especially relevant in South Asian settings, where vegetarian dietary practices are common, and functional deficiency can co-occur with hyperhomocysteinemia.^(60–62)^ Evidence from Indian cohorts (e.g. the Pune Maternal Nutrition Study) links low vitamin B_12_ and functional deficiency markers (including methylmalonic acid [MMA]) with higher offspring insulin resistance, supporting a life-course role of one-carbon metabolism in cardiometabolic risk.^(63,64)^ Functionally, MMA and homocysteine can complement serum B_12_ to capture functional B_12_ insufficiency, strengthening risk stratification in settings where dietary exposure patterns differ markedly across Asian subregions.^(65,66)^ Finally, iodine status – highly salient in parts of Asia due to seafood/seaweed exposures and salt iodization variability – interfaces with thyroid hormone biology and can influence lipid metabolism; emerging reviews also highlight metabolomic shifts under iodine perturbation.^(67,68)^

### Fatty acid profiles as diet-responsive metabolomic readouts

Fatty acid profiles provide diet-responsive metabolomic readouts that capture both dietary fat exposure and endogenous remodelling, with clear relevance for Asian populations, where staple diets and fat sources vary widely. The circulating and erythrocyte membrane eicosapentaenoic acid (EPA) + docosahexaenoic acid (DHA) (the Omega-3 Index) reflect longer-term marine n−3 exposure and have been proposed as risk-relevant indices for coronary outcomes, while mechanistic and clinical literature supports the anti-inflammatory and pro-resolving actions of marine n−3 fatty acids.^(69,70)^ Importantly, fatty acid signatures can inform precision nutrition not only through ‘intake-status’ markers (e.g. EPA/DHA) but also through endogenous pathway markers that link carbohydrate-heavy diets to lipid dysregulation. Excess carbohydrates can be converted in the liver into palmitate (16:0) and desaturated to palmitoleate (16:1n–7); accordingly, de novo lipogenesis (DNL) proxy indices derived from very LDL-triacylglycerol fatty acid patterns have been used to approximate hepatic lipogenic remodelling and tend to increase under high-carbohydrate feeding, alongside increases in plasma triglycerides.^(71–73)^ However, these proxies should be interpreted in the context of the diet (e.g. palm-oil-derived palmitate) and, where feasible, complemented by postprandial designs or hepatic-output–enriched fractions.^(74)^ In practice, combining omega-3 status markers with DNL-related signatures can help distinguish individuals, whose risk biology is driven more by fat quality versus carbohydrate-to-fat conversion, thereby supporting culturally tailored recommendations in Asian populations.

### Population-specific metabolomic signatures

Metabolomic fingerprints vary considerably across Asian subpopulations due to differences in genetic background, gut microbiome composition, habitual dietary substrates (including staple carbohydrates, fermentation practices, and regional fat sources), and environmental exposure.^(18,75,76)^ Importantly, these differences extend beyond lipid- and amino-acid patterns to include microbiome-derived metabolites (e.g. SCFAs, bile-acid derivatives, and TMAO),^(51,54,56)^ carbohydrate-related markers of glycemic stress,^(41,44–46)^ and fatty acid profiles reflecting both intake (omega-3/omega-6 balance) and endogenous remodelling (e.g. DNL-related signatures).^(69–74)^ In some settings, these metabolite signatures can be further contextualized by micronutrient-related functional indicators that track one-carbon metabolism (e.g. vitamin B12–related MMA/homocysteine patterns).^(60,63,65)^

- **East Asians** often exhibit metabolite–nutrient interaction patterns at the population level, including phospholipid and amino acid signatures that track rice-and fermented food-rich diets; these patterns may coincide with microbiome-derived metabolite differences.^(76,77)^
- **South Asians** exhibit characteristic elevations in branched-chain amino acids (BCAAs) and acylcarnitines in multi-ethnic analyses, consistent with early metabolic inflexibility and mitochondrial substrate-handling differences.^(75,78,79)^ In subgroups, functional vitamin B_12_ insufficiency and elevated homocysteine levels may further modify risk profiles, aligning with long-standing vegetarian dietary traditions in parts of South Asia.^(60,64)^
- **Southeast Asians** can display distinct lipidomic and fatty acid features in multi-ethnic settings, and regional culinary fat sources (e.g. coconut- and palm-based oils) have been evaluated in human studies for their effects on cardiometabolic markers,^(80–82)^ requiring careful metabolomic interpretation to distinguish dietary saturated fat intake from endogenous, carbohydrate-driven DNL where relevant.^(74)^

These population-specific patterns highlight the need to establish regionally appropriate reference ranges rather than relying on Western-centric metabolomic interpretations of the data. Table 1 summarizes the key metabolomic and genomic biomarkers that are particularly relevant to precision nutritional approaches in Asian populations.

**Table 1.** Key metabolomic and genomic biomarkers relevant to precision nutrition in Asian populations Table 1 long description.

Biomarker category	Representative biomarkers	Mechanistic role	Relevance in Asian populations
Lipid-derived metabolites	Lysophosphatidylcholines (lysoPCs), phosphatidylcholines	Reflect oxidative stress, membrane turnover, endothelial dysfunction	Elevated in metabolically unhealthy overweight Koreans; associated with visceral adiposity and early insulin resistance
Carbohydrate-related metabolites	1,5-anhydroglucitol (1,5-AG), lactate, α-hydroxybutyrate (α-HB), mannose, methylglyoxal/3-deoxyglucosone, sorbitol/fructose (polyol pathway)	Postprandial glycemic stress, reduced metabolic flexibility, glycation/carbonyl stress and redox imbalance under sustained hyperglycaemia	Particularly relevant where refined grains/high glycemic-load staples are common and β-cell reserve may be comparatively limited; supports identification of ‘carbohydrate responders’
Micronutrient-related biomarkers	Retinal/retinoic acid (vitamin A derivatives), methylmalonic acid (MMA), homocysteine (functional B_12_ status), iodine-related axis (often assessed alongside metabolomics)	Nutrient status tied to actionable biology (retinoid signalling, one-carbon metabolism, thyroid axis influencing lipid handling)	Vitamin A–linked metabolites reported as early predictors in Korean adults; B_12_ functional insufficiency relevant in some South Asian vegetarian subgroups; iodine axis salient where seafood/seaweed intake and iodization policies vary
Amino acids and acylcarnitines	BCAAs, aromatic amino acids, C2–C18 acylcarnitines	Indicate mitochondrial β-oxidation efficiency and metabolic flexibility	South Asians often show elevated BCAAs linked to early insulin resistance and ectopic fat accumulation
Oxidative-stress markers	8-epi-PGF_2α_, oxidized LDL	Capture reactive oxygen species burden and vascular injury	Strong predictors of metabolic syndrome and obesity phenotypes in Korean cohorts
Microbiome-derived metabolites	Short-chain fatty acids (SCFAs: acetate, propionate, butyrate), trimethylamine N-oxide (TMAO), bile-acid derivatives	SCFAs linked to insulin sensitivity, TMAO and bile-acid signalling (FXR/TGR5) relate to cardiometabolic pathways	Captures the high diversity of Asian fermented food/fibre intake; supports mechanistic linkage between cultural diet and metabolic phenotypes
Fatty acid profiles	Omega-3 Index (EPA + DHA), omega-6 : omega-3 balance, palmitate/palmitoleate-related signatures (DNL proxies)	Dietary fat exposure and endogenous remodelling; inflammatory tone; carbohydrate-to-fat conversion (hepatic lipogenesis)	Relevant given regional differences in fish/seafood intake and culinary oils; helps distinguish ‘fat-quality–driven’ vs ‘carbohydrate-to-fat conversion–driven’ risk biology
Genetic variants (GRS components)	*TMEM182, NPC1L1, APOA5, CETP*, *KCNQ1, TCF7L2, SLC30A8, FADS1/2, BCMO1, FUT2/6* and related loci	Influence adipogenesis, cholesterol absorption, triglyceride turnover, HDL remodelling, β-cell function/glycaemia, PUFA biosynthesis, provitamin A conversion, and host–microbe/nutrient-handling pathways	Asian-enriched or ancestry-informative loci can shape susceptibility at lower BMI and interact with culturally patterned exposures (high glycemic staples, fermentation, fat sources)
Integrated omics scores	Genetic risk scores combined with metabolite panels	Integrate inherited and dynamic risk	Improve prediction of metabolic syndrome and type 2 diabetes compared with genetic or clinical markers alone

## Dietary phenotypes and nutrient–metabolite mechanisms in Asian populations

Dietary exposure in Asian populations is not defined solely by macronutrient composition but by culturally embedded patterns that shape staple food selection, preparation techniques, fermentation practices, meal structure, and habitual food combinations. These dietary phenotypes influence glycemic load, fatty acid balance, sodium intake, fibre availability, and phytochemical exposure, thereby generating population-specific metabolomic and microbiome configurations. In carbohydrate-dominant dietary settings common across East and South Asia, metabolomics provides mechanistic insight into how these culturally patterned nutrient exposures translate into lipidomic shifts, oxidative stress signatures, and alterations in amino acid metabolism.^(51,83,84)^ Therefore, understanding dietary phenotypes at the pattern level is essential before interpreting nutrient-level mechanisms.^(2)^ Pattern-level exposures provide the ecological context in which nutrient–metabolite interactions unfold.

### Fish- and seafood-rich dietary patterns

Fish and seafood consumption is culturally central in many coastal East and Southeast Asian populations, often representing long-term habitual exposure rather than occasional intake. Marine-derived long-chain n−3 PUFAs, particularly EPA and DHA, modulate hepatic triglyceride synthesis, membrane phospholipid remodelling, and inflammatory resolution pathways.^(85,86)^ Lipidomic profiling demonstrates that dietary n−3 intake is reflected in circulating triglyceride and phosphatidylcholine species, influencing downstream insulin signalling and inflammatory mediator balance.^(87)^

However, metabolic consequences depend substantially on preparation practices. Salted, dried, or deep-fried seafood products may increase sodium intake^(88)^ and introduce oxidized lipid species, potentially attenuating cardiometabolic benefits.^(89)^ Precision-nutrition frameworks in Asian contexts should therefore differentiate fresh marine-based dietary patterns from highly processed seafood preparations when interpreting lipidomic and inflammatory biomarkers.

### Soy and traditional soy-based dietary exposures

Soybeans and traditional soy-based foods, including tofu, soy milk, tempeh, miso, doenjang, and natto, are culturally salient protein sources in East Asia. Epidemiological and clinical studies indicate that soy intake is associated with improved lipid profiles and reduced cardiovascular risk markers.^(90,91)^ Isoflavones, such as genistein and daidzein, influence endothelial function, lipid metabolism, and insulin sensitivity through oestrogen receptor–mediated transcriptional pathways and antioxidant effects.^(90)^

A defining feature of soy in precision nutrition is interindividual variability in isoflavone metabolism. Gut microbial conversion of daidzein to equol varies widely across individuals and populations.^(92)^ Equol production status has been associated with differential metabolic and vascular responses to soy intake.^(93)^ Integration of dietary data with microbiome and metabolomic profiling thus enables the stratification of responders and non-responders within culturally consistent soy-based dietary patterns, illustrating how identical food exposure can generate divergent metabolomic phenotypes.

### Plant-forward dietary patterns and carbohydrate quality

Many traditional Asian dietary patterns are plant-forward and characterized by a substantial intake of grains, vegetables, and legumes. When minimally processed, these diets provide dietary fibre and polyphenols that promote microbial fermentation and SCFA production.^(51,94,95)^ SCFAs bind to GPR41 and GPR43 and enhance insulin sensitivity and reduce hepatic lipogenesis,^(84)^ and stimulate glucagon-like peptide-1 (GLP-1) secretion.^(96)^

However, carbohydrate quality and degree of refinement critically shape metabolic outcomes. Diets dominated by polished white rice and refined wheat products may increase postprandial glycemic excursions and exacerbate β-cell stress, particularly in Asian populations with reduced β-cell reserve.^(4,97)^ Metabolomic studies have linked high glycemic-load dietary patterns to elevations in BCAAs, acylcarnitines, and lipid species associated with impaired mitochondrial β-oxidation and early insulin resistance.^(98,99)^ Thus, the classification of diets as ‘plant-based’ without accounting for processing level and meal composition may obscure important metabolic heterogeneity.

### Nutrient–Metabolite mechanisms within cultural contexts

Within these broader dietary phenotypes, specific nutrient-level mechanisms provide mechanistic insights into how culturally congruent modifications can improve metabolic resilience.

- **Dietary fibre and fermentation-derived metabolites.** Soluble fibre is fermented by gut microbiota to generate SCFAs, including acetate, propionate, and butyrate, which regulate hepatic lipid metabolism, immune signalling, and insulin sensitivity.^(51)^ In Korean metabolomics studies, higher fibre intake has been associated with increased fatty acid β-oxidation, reduced lipid peroxidation, and improved phospholipid balance.^(36,40)^ These findings suggest that increasing fibre intake within rice-dominant dietary contexts may partially counteract oxidative and inflammatory metabolic phenotypes.
- **Legume substitution and glycemic modulation.** Legumes, including soybeans, lentils, and chickpeas, are traditional components of many Asian dietary patterns and represent culturally feasible substitution strategies. Randomized controlled trials (RCTs) in Korean women have demonstrated that replacing one-third of daily rice intake with legumes reduces oxidative stress biomarkers, increases adiponectin concentrations, and promotes modest weight reduction.^(100,101)^ Mechanistically, these changes are associated with enhanced AMP-activated protein kinase-mediated fatty acid oxidation and improved antioxidant capacity.^(102)^ Such culturally aligned substitutions illustrate how traditional foods can be leveraged within precision nutrition frameworks to mitigate glycemic and lipid-related risk.

### Conceptual integration

Collectively, fish-rich, soy-centred, and plant-forward dietary phenotypes generate distinct nutrient–metabolite configurations that shape cardiometabolic risk trajectories in Asian populations. By explicitly linking staple foods, preparation methods, and fermentation practices to candidate omics biomarkers, such as lipidomic signatures, SCFAs, amino acid profiles, and oxidative-stress indicators, precision-nutrition approaches can move beyond generalized dietary advice toward culturally embedded, mechanism-informed personalization strategies.

## Integrated genetic–metabolomic risk models

Multi-omics integration provides a refined understanding of metabolic risk by combining genetic predisposition and dynamic metabolomic states. In Asian populations, where specific genetic variants and characteristic metabolic phenotypes frequently co-occur, integrated models offer substantial advantages over single-parameter assessments.

### Genetic variants enriched in Asian populations

GWASs conducted in Korean, Chinese, and broader Asian cohorts have identified population-enriched variants that influence adiposity, lipid metabolism, and glycemic regulation.^(21,103–105)^ Variants in *TMEM182, NPC1L1, APOA5*, and *CETP* affect adipogenesis, cholesterol absorption, triglyceride metabolism, and high-density lipoprotein (HDL) remodelling.^(19,20,106,107)^ These variants interact with nutrient-sensitive transcriptional regulators, including peroxisome proliferator-activated receptors (PPARα/γ), sterol regulatory element-binding proteins, and the liver X receptor, thereby shaping metabolic responses to dietary fats and high-glycemic carbohydrates.^(58,108)^

In East Asian populations, genetic risk scores (GRSs) incorporating population-enriched variants have demonstrated improved risk discrimination for cardiometabolic outcomes, such as type 2 diabetes, compared with scores derived from European datasets.^(109,110)^ In addition, weighted GRS approaches that account for variant-specific effect sizes have been shown to further enhance predictive accuracy in Asian cohorts,^(22,23)^ supporting the use of ancestry-informed weighting and calibration for risk stratification.

### Metabolomic biomarkers as dynamic indicators

Metabolomic profiles complement genetic data by capturing dynamic and time-sensitive metabolic perturbations. Elevated lysoPC concentrations reflect phospholipid turnover, mitochondrial stress, and systemic inflammation.^(37,38)^ Altered amino acid signatures, including BCAAs, aromatic amino acids, and acylcarnitines, provide insights into mitochondrial β-oxidation efficiency, nitrogen metabolism, and microbiome–host interactions.^(111,112)^ Oxidative stress markers such as 8-epi-PGF_2α_ offer an additional dynamic layer that reflects lifestyle, environmental exposure, and nutritional status.^(9,22)^

### Synergistic value of integrated models

Studies in Korean cohorts have demonstrated that genetic–metabolomic integration improves the prediction of metabolic syndrome, type 2 diabetes, and obesity phenotypes.^(9,21–23)^ Multi-omics models capture the interactions between inherited susceptibility and modifiable metabolic conditions, enabling the precise identification of individuals likely to benefit from specific dietary interventions. For example, individuals with a high GRS for dyslipidemia may respond more favourably to legume substitution or reduced glycemic load when their metabolomic profiles concurrently exhibit markers of phospholipid dysregulation or heightened oxidative stress.^(6,22,108)^

These findings underscore the value of moving beyond single-biomarker frameworks toward integrated, mechanistically informed risk assessments.

## Translational framework for omics-guided precision nutrition in Asia

**Figure 2.**
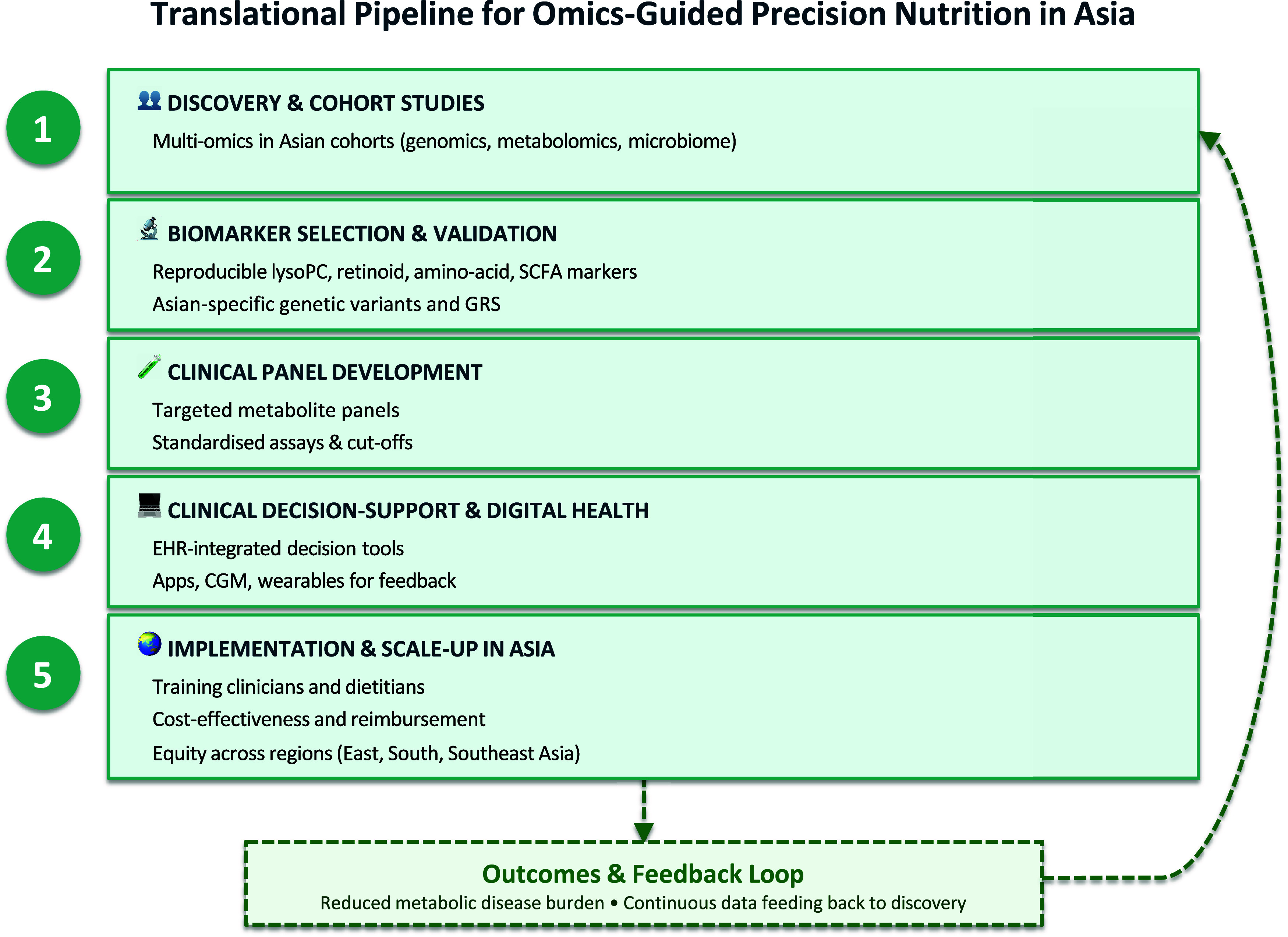
Figure 2 long description. Translational pipeline for omics-guided precision nutrition in Asia. The pipeline begins with discovery omics studies in Asian cohorts, followed by the validation and standardization of biomarker panels. Clinically feasible assays and decision support tools are then integrated into healthcare and digital health platforms. Implementation and scale-up efforts must address workforce training, cost-effectiveness, and equity to ensure that the benefits of precision nutrition are accessible across diverse Asian populations.

Translating multi-omics findings into clinical and public health practices requires the coordinated development of analytical infrastructure, workforce capacity, technological tools, and equity considerations. Asian countries vary widely in terms of laboratory resources, healthcare systems, and dietary environments, necessitating regionally adaptive approaches. A stepwise translational pipeline for omics-guided precision nutrition in Asia is shown in Figure 2.

### Analytical standardization and feasibility

A major barrier to the clinical adoption of metabolomics is the lack of cross-platform standardization.^(113,114)^ Variability in sample processing, analytical platforms, and data interpretation limits the comparability of studies.^(115–117)^ The development of standardized, clinically validated targeted metabolite panels offers a feasible solution. Such panels should prioritize biomarkers with established mechanistic relevance in Asian populations, including lysoPC species, retinoid metabolites, amino acid ratios, and oxidative stress markers.^(9)^

### Clinician training and decision-support systems

The effective use of omics data requires clinicians to understand metabolic pathways, gene–diet interactions, and biomarker interpretation. However, surveys indicate that many healthcare professionals report limited confidence in applying omics data to nutritional decision-making.^(118,119)^ Integrating omics education into medical and dietetics curricula, coupled with decision-support systems embedded within electronic health records, can reduce interpretive barriers and support the consistent implementation of precision nutrition.^(120,121)^

### Digital-health integration

Wearable biosensors, CGM, mobile diet-tracking applications, and artificial intelligence (AI)-based diet analysis tools offer scalable platforms for personalized nutrition.^(120–122)^ Emerging microbiome-enabled extensions of these platforms now include app-linked, at-home gut microbiome testing with standardized reporting pipelines, enabling microbiome features to be incorporated into digital precision-nutrition workflows.^(123,124)^ These technologies can incorporate omics-derived biomarkers to generate adaptive dietary recommendations that are updated in response to real-time metabolic feedback. In particular, AI-driven microbiome–diet response prediction models that integrate gut microbiome profiles with dietary records and CGM data have been used to predict individualized postprandial responses and to support algorithm-guided dietary recommendations.^(6,8)^ Digital feedback systems can also integrate microbiome-derived metabolites (e.g. SCFAs and related host–microbe co-metabolism signatures) to provide actionable, phenotype-linked coaching aligned with culturally patterned exposures such as fermented foods and variable fibre sources.^(51,122)^ Evidence and clinical validation levels vary across platforms, highlighting the need for harmonized standards and rigorous evaluation before broad implementation. Given current variability in consumer-facing microbiome testing, implementation should emphasize analytical standardization, clinical validation, privacy protection, and equitable access.^(123,124)^ Digital health integration is particularly promising in Asia, where mobile technology adoption is high and health systems are increasingly embracing telehealth.^(125–127)^

### Equity and regional diversity

Despite these rapid advances, omics research in Asia remains concentrated in Korea, Japan, and China. South and Southeast Asian populations are underrepresented despite their high metabolic disease burden and diverse dietary exposure. Limited laboratory capacity, socioeconomic barriers, and variable access to healthcare resources further challenge the equitable implementation.^(128–132)^ Ensuring affordability, cultural compatibility, and adequate representation in omics datasets is essential to prevent the widening of metabolic health disparities.

## Future research priorities

Advancing precision nutrition in Asian populations requires strategic investments in multiple research areas. Several high-priority areas have emerged from recent evidence.

### Longitudinal multi-omics cohorts

First, there is a clear need for longitudinal cohort studies that track metabolomic, genomic, microbiomic, and proteomic changes over time.^(114,133–136)^ Such studies will help determine the stability of metabolomic signatures, identify early biochemical shifts preceding metabolic deterioration, and clarify temporal relationships among diet, metabolic pathways, and disease risk. Continuous or repeated sampling is particularly important in Asian populations, where glycemic responses and oxidative stress can fluctuate dramatically according to dietary composition and environmental exposure.^(6,8)^

### Systems-level integration of omics datasets

Integrating genomics, metabolomics, and microbiome data into cohesive system-level models will enable deeper mechanistic insights into nutrient–metabolite interactions.^(9,137,138)^ Computational approaches, such as network modelling, machine learning, and causal inference frameworks, can help map dietary exposures onto biological pathways and identify interaction nodes that may serve as targets for tailored dietary interventions.^(139,140)^

## Representation of under-studied Asian populations

Future research should expand beyond East Asian cohorts to include South and Southeast Asian populations, who face disproportionately high metabolic disease burdens.^(15,97,141)^ These populations often exhibit early metabolic impairment, unique fat distribution patterns, and distinct dietary exposures that are not adequately represented in current omics datasets. Increasing the representation improves the accuracy, generalizability, and cultural relevance of precision nutrition models.^(128)^

### Randomized controlled trials using omics endpoints

There is a critical need for dietary intervention RCTs that incorporate omics biomarkers as exposure measures and mechanistic outcomes.^(6,11)^ As a key example, a randomized, open-label trial conducted in China demonstrated that baseline gut microbiome features could predict individualized glycemic benefits from dietary fibre supplementation in adults with prediabetes, supporting microbiome-informed responder stratification for precision nutrition.^(142)^ Building on this emerging evidence, future RCTs in Asian populations should prioritize culturally relevant and scalable interventions, with a focus on fermentable dietary fibres, legumes, soy-based foods, omega-3–rich fish and seafood, and fermented foods, paired with multi-omics endpoints (e.g. microbiome features, SCFAs, metabolomics, and postprandial glycemic readouts such as CGM) to identify biological responders versus non-responders and to define minimal effective ‘doses’ of dietary modification. Such trials can provide mechanistic evidence necessary for personalized dietary guidelines.^(8)^

In addition to published efficacy trials, protocol papers and trial registries of ongoing or planned studies can provide timely evidence on emerging study designs, research priorities, and upcoming precision-nutrition interventions in Asian populations. For example, the New Zealand SYNERGY study – a fully diet-controlled, parallel, residential intervention conducted in Asian Chinese and European Caucasian adults with prediabetes – illustrates the value of controlled feeding designs for isolating diet effects while enabling cross-population comparisons without weight-loss confounding.^(143)^

### Implementation science and real-world application

Finally, implementation-science frameworks are needed to evaluate the feasibility, cost-effectiveness, and long-term sustainability of omics-guided dietary interventions in diverse healthcare settings.^(131,144,145)^ These frameworks should address practical factors, including laboratory capacity, digital health integration, clinician training, reimbursement structures, and patient acceptability. The major research and implementation priorities for omics-guided precision nutrition in Asia are summarized in Table 2.

**Table 2.** Research and implementation priorities for omics-guided precision nutrition in Asian populations Table 2 long description.

Priority area	Specific objective	Target population/settings	Expected contribution to precision nutrition
Longitudinal multi-omics cohorts	Track temporal changes in metabolome, genome, microbiome, and clinical outcomes	East, South, and Southeast Asian adults at varying metabolic risk	Identify early molecular signatures preceding metabolic deterioration; refine risk prediction models
Systems-level multi-omics integration	Combine genomics, metabolomics, and microbiome data using network and machine-learning approaches	Research consortia and academic centres	Map nutrient–metabolite pathways and interaction nodes that can be targeted by dietary interventions
Inclusion of under-represented populations	Expand recruitment beyond East Asia to South and Southeast Asia	Rural and urban communities with high metabolic-disease burden	Improve generalizability and cultural relevance of precision-nutrition guidelines
Omics-informed randomized controlled trials	Test dietary patterns (e.g. legume substitution, higher fibre, Asian-adapted Mediterranean diets) using omics endpoints	Individuals with obesity, dyslipidemia, prediabetes	Distinguish responders vs non-responders; determine minimal effective dietary changes
Implementation science and health-system integration	Evaluate feasibility, cost-effectiveness, and scalability of omics-guided nutrition care	Primary care, hospitals, community clinics	Inform reimbursement models, workflow integration, and sustainable service delivery
Digital-health and decision-support tools	Embed omics-based risk scores into clinical decision-support and consumer-facing apps	Clinicians, dietitians, and patients using CGM/wearables	Enable near real-time feedback and adaptive personalized dietary recommendations
Capacity building and equity	Develop training programmes and ensure affordable access to omics tests	Healthcare professionals and policy makers across Asian regions	Reduce knowledge gaps and prevent widening of metabolic health disparities

## Conclusion

Precision nutrition provides a promising approach for improving metabolic health across Asia by integrating genetic, metabolomic, microbiome, and environmental data into personalized dietary recommendations. The Asian populations are characterized by distinct metabolic phenotypes, including heightened visceral adiposity, reduced β-cell reserve, increased oxidative stress, and culturally shaped dietary exposures, which influence susceptibility to metabolic diseases. Multi-omics biomarkers, such as lysoPCs, retinoid metabolites, amino acid signatures, oxidative stress indicators, and emerging markers of postprandial glycemic excursions, microbiome activity, and fatty acid remodelling, offer sensitive tools for the early detection of metabolic dysfunction and may guide tailored dietary interventions.

Genetic–metabolomic integration further enhances risk stratification by accounting for both inherited susceptibility and dynamic metabolic states. Translating these insights into practice will require robust analytical standardization, improved clinician training, digital health solutions capable of supporting adaptive personalized guidance, and a commitment to equity across diverse Asian regions. Future progress will also depend on longitudinal multi-omics cohorts and omics-endpoint dietary RCTs, complemented by protocol papers and trial registries that provide early visibility into ongoing and upcoming interventions. By aligning mechanistic evidence with culturally informed dietary strategies, omics-guided precision nutrition has the potential to contribute meaningfully to early disease prevention and reduce the growing burden of metabolic disorders in Asia.

## Figure 1. Long description

The diagram illustrates the multi-omics precision nutrition framework for Asian populations. It starts with distinct Asian metabolic features such as increased visceral adiposity, reduced beta-cell reserve, and cultural dietary patterns. This leads to multi-omics profiling, which includes genomics, metabolomics, microbiome, and oxidative stress markers. The nutrient-metabolite mechanisms section describes how dietary components like fiber, legumes, refined grains, and oils affect metabolic processes. The integrated genetic-metabolomic risk models section explains the use of genetic risk scores and metabolite panels for risk classification and early detection of metabolic dysfunction. Finally, the precision nutrition interventions in Asia section outlines personalized dietary patterns, omics-informed control, digital health integration, and culturally adapted strategies.

Navigate back to Figure 1..

## Table 1. Long description

The table presents key metabolomic and genomic biomarkers relevant to precision nutrition in Asian populations, emphasizing the need for regionally appropriate reference ranges. It includes categories such as lipid-derived metabolites, carbohydrate-related metabolites, micronutrient-related biomarkers, amino acids and acylcarnitines, oxidative-stress markers, microbiome-derived metabolites, fatty acid profiles, genetic variants, and integrated omics scores. Each category lists representative biomarkers, their mechanistic roles, and their relevance in Asian populations. For example, lipid-derived metabolites like lysophosphatidylcholines are elevated in metabolically unhealthy overweight Koreans and are associated with visceral adiposity and early insulin resistance. Carbohydrate-related metabolites such as 1,5-anhydroglucitol and lactate reflect postprandial glycemic stress and reduced metabolic flexibility. The table also highlights the importance of micronutrient-related biomarkers like vitamin A derivatives and methylmalonic acid, which are tied to nutrient status and actionable biology. Amino acids and acylcarnitines indicate mitochondrial beta-oxidation efficiency, while oxidative-stress markers capture reactive oxygen species burden and vascular injury. Microbiome-derived metabolites like short-chain fatty acids are linked to insulin sensitivity and bile-acid signaling. Fatty acid profiles, genetic variants, and integrated omics scores further contribute to understanding dietary fat exposure, endogenous remodelling, and inherited risk. The table underscores the diversity of Asian populations and the need for tailored nutritional approaches.

Navigate back to Table 1..

## Figure 2. Long description

The flowchart outlines the translational pipeline for omics-guided precision nutrition in Asia. It starts with discovery and cohort studies involving multi-omics in Asian cohorts, including genomics, metabolomics, and microbiome analysis. The second stage focuses on biomarker selection and validation, identifying reproducible markers such as lysoPC, retinoid, amino-acid, SCFA markers, and Asian-specific genetic variants and GRS. The third stage involves clinical panel development, creating targeted metabolite panels and standardized assays and cut-offs. The fourth stage integrates clinical decision-support and digital health tools, including EHR-integrated decision tools, apps, CGM, and wearables for feedback. The final stage emphasizes implementation and scale-up in Asia, addressing training for clinicians and dietitians, cost-effectiveness, reimbursement, and equity across regions such as East, South, and Southeast Asia. The process includes an outcomes and feedback loop to reduce metabolic disease burden and continuously feed data back into discovery.

Navigate back to Figure 2..

## Table 2. Long description

The table outlines research and implementation priorities for omics-guided precision nutrition in Asian populations. It consists of four columns: Priority area, Specific objective, Target population/settings, and Expected contribution to precision nutrition. The table has six rows, each detailing specific objectives, target populations, and expected contributions. The priority areas include longitudinal multi-omics cohorts, systems-level multi-omics integration, inclusion of under-represented populations, omics-informed randomized controlled trials, implementation science and health-system integration, digital-health and decision-support tools, and capacity building and equity. Each row provides specific objectives, target populations or settings, and the expected contributions to precision nutrition. For example, the first row focuses on tracking temporal changes in metabolome, genome, microbiome, and clinical outcomes among East, South, and Southeast Asian adults at varying metabolic risk, aiming to identify early molecular signatures preceding metabolic deterioration and refine risk prediction models.

Navigate back to Table 2..
